# A commentary on ‘Auxiliary use of ChatGPT in surgical diagnosis and treatment’ (Int J Surg 109 (2023) 3940–3943)

**DOI:** 10.1097/JS9.0000000000001126

**Published:** 2024-01-18

**Authors:** Ziwei Li, Yan Shu, Yongzhong Tang, Weiju Lai

**Affiliations:** Chongqing University FuLing Hospital, Chongqing, People’s Republic of China

*Dear Editor*,

The emergence of ChatGPT has sparked a revolution across various industries worldwide, leveraging its remarkable capabilities in information ingestion, processing, and learning. ChatGPT has found extensive applications in numerous domains, exhibiting unmatched prowess in information management, surpassing the capabilities of the human brain. In this interesting review authored by MD Kahei Au, the significant role of ChatGPT in surgical diagnosis and treatment is emphasized, highlighting its potential to revolutionize the clinical care paradigm. MD Kahei Au also pointed out the potential issues and risks associated with its implementation in surgical treatment. However, further interpretation is warranted for certain aspects discussed in this article.

Firstly, the citation of the ninth reference is inaccurate as it represents a diagnostic case study rather than a complete clinical trial, and it was conducted retrospectively^1^. The claim that artificial intelligence (AI) has been proven to improve clinical decision-making and reduce the risk of patient harm based on a single case is highly inaccurate. In fact, some report that ChatGPT sometimes not only provides absurd answers but even fabricates literature to support its claims^2^.

Secondly, ChatGPT is fundamentally a conversational AI rather than possessing in-depth analytical capabilities^3^. The understanding of patients comes from the summation of information provided by doctors. However, in clinical practice, patients’ emotions, facial expressions, postures, communication patterns, and other information cannot be fully captured by AI. Otherwise, many clinical examinations and medical history records are subjective in nature and cannot be presented to AI in a data-driven format for learning and analysis. Relying on incomplete data analysis results provided by AI, doctors may face serious consequences.

Moreover, the establishment of clinical efficacy for ChatGPT demands considerable ethical considerations and data analysis efforts. In contrast to conventional clinical trials, ChatGPT functions as an open-source AI system, leading to temporal limitations on its efficacy and accuracy^4^. As the field of medicine is constantly evolving and advancing, and new research and discoveries may impact previous responses to AI. The incorporation of AI in clinical practice may necessitate the utilization of specifically tailored experimental protocols.

In conclusion, clinical diagnosis and decision-making is a dynamic process of comprehensive assessment. However, for AI systems, acquiring comprehensive and accurate information and providing suggestions that bridge the gap between the virtual and real world remains a formidable challenge^5^. Given the intricate and time-critical aspects of surgical diagnosis and treatment, ChatGPT’s existing framework may be better suited for analyzing real-time outcomes and digitized data in disciplines such as radiology, pathology, and laboratory medicine.

## Ethical approval

None.

## Consent

None.

## Sources of funding

Chongqing Medical Scientific Research Project (Joint project of Chongqing Health Commission and Science and Technology Bureau) (2020FYYX189).

## Author contribution

L.Z.: writing – original draft; S.Y.: investigation; L.W.: supervision; T.Y.: writing – review and editing.

## Conflicts of interest disclosure

There are no conflicts of interest.

## Research registration unique identifying number (UIN)

None.

## Guarantor

Tang Yongzhong.

## Data availability statement

Data sharing is not applicable to this article as no new data were created or analyzed in this study.

## Provenance and peer review

None.
